# Comparative bibliometric analysis of artificial intelligence-assisted polyp diagnosis and AI-assisted digestive endoscopy: trends and growth in AI gastroenterology (2003–2023)

**DOI:** 10.3389/fmed.2024.1438979

**Published:** 2024-09-18

**Authors:** Ziye Peng, Xiangyu Wang, Jiaxin Li, Jiayi Sun, Yuwei Wang, Yanru Li, Wen Li, Shuyi Zhang, Ximo Wang, Zhengcun Pei

**Affiliations:** ^1^Medical School, Tianjin University, Tianjin, China; ^2^Department of Endoscopy, Tianjin Union Medical Center, Tianjin, China; ^3^Tianjin Third Central Hospital, Tianjin, China

**Keywords:** artificial intelligence, polyp, endoscopy, bibliometric analysis, research trend

## Abstract

**Introduction:**

Artificial intelligence is already widely utilized in gastroenterology. This study aims to comprehensively evaluate the research hotspots and development trends within the field of AI in gastroenterology by employing bibliometric techniques to scrutinize geographical distribution, authorship, affiliated institutions, keyword usage, references, and other pertinent data contained within relevant publications.

**Methods:**

This investigation compiled all pertinent publications related to artificial intelligence in the context of gastrointestinal polyps and digestive endoscopy from 2003 to 2023 within the Web of Science Core Collection database. Furthermore, the study harnessed the tools CiteSpace, VOSviewer, GraphPad Prism and Scimago Graphica for visual data analysis. The study retrieved a total of 2,394 documents in the field of AI in digestive endoscopy and 628 documents specifically related to AI in digestive tract polyps.

**Results:**

The United States and China are the primary contributors to research in both fields. Since 2019, studies on AI for digestive tract polyps have constituted approximately 25% of the total AI digestive endoscopy studies annually. Six of the top 10 most-cited studies in AI digestive endoscopy also rank among the top 10 most-cited studies in AI for gastrointestinal polyps. Additionally, the number of studies on AI-assisted polyp segmentation is growing the fastest, with significant increases in AI-assisted polyp diagnosis and real-time systems beginning after 2020.

**Discussion:**

The application of AI in gastroenterology has garnered increasing attention. As theoretical advancements in AI for gastroenterology have progressed, real-time diagnosis and detection of gastrointestinal diseases have become feasible in recent years, highlighting the promising potential of AI in this field.

## 1 Introduction

Gastrointestinal polyps serve as precursors to a multitude of digestive ailment (1–4). Colorectal polyps possess the potential to transition into colorectal cancer over time, whereas gastric polyps are capable of leading to gastric cancer, gastric ulcers, and chronic atrophic gastritis (5–7).

Digestive endoscopy stands as an indispensable tool for both the identification of suspicious lesions and the execution of minimally invasive procedures in the various segments of the gastrointestinal tract (8). It plays a pivotal role in both the diagnosis and treatment of gastrointestinal maladies (9). Thanks to advancements in electronic endoscopes, optical probes, and robot-assisted technology, digestive endoscopy has achieved substantial progress in fields including minimally invasive treatments, early tumour diagnosis, precision medicine, and therapeutic efficacy (10, 11). These enhancements have significantly elevated the quality of life for patients, accomplishing this by mitigating discomfort, hastening recovery, and supplying more precise diagnoses and targeted interventions (12, 13).

Artificial intelligence (AI) has undergone rapid development and has found wide-ranging application across various domains in recent years (14–16). The core objective underlying the integration of AI into the realm of medicine is to streamline clinical decision-making, curtail medical oversights, and augment medical efficiency (14). With the support of AI, digestive endoscopy technology has grown even more potent, showcasing the capacity to execute tasks such as automatic identification of gastrointestinal polyps, heightening the quality of endoscopic images, and forecasting the depth of cancer infiltration (17–19). AI has witnessed extensive adoption in the domain of gastrointestinal polyp diagnosis, encompassing tasks like gastrointestinal polyp detection, segmentation, classification, and the deployment of real-time polyp detection systems (20, 21). The AI-assisted diagnosis of gastrointestinal polyps has emerged as a substantial and vital avenue of research within the domain of AI digestive endoscopy. Drawing from the corpus of articles catalogued within the Web of Science’s core database, it becomes apparent that there have been more than 100 publications each year pertaining to digestive AI in gastrointestinal polyps since 2022. Furthermore, there exists a steady upswing in the number of articles issued within the field of AI in digestive endoscopy over the previous 3 years (22, 23). These trends denote an accelerated development within the domain, underscoring the need for researchers to remain abreast of the latest advancements.

This method of objective assessment empowers researchers to gain insights into research inclinations, the academic contributions of different teams and nations, and the significant scholars within a given discipline. Consequently, this study employs bibliometrics to scrutinize pertinent details encompassing various nations, authors, academic institutions, periodicals, references, citations, developmental patterns, and research emphases in the domain of gastrointestinal polyps and AI over the 20-year interval spanning from 2003 to 2023. Through the execution of this analysis, our aspiration is to equip researchers with a comprehensive understanding of the evolution and the present research landscape in this domain over the preceding two decades. Simultaneously, we aim to assist scholars in pinpointing areas of research concentration and in forecasting forthcoming trends.

## 2 Methods

### 2.1 Data source and search strategies

**Figure 1:**
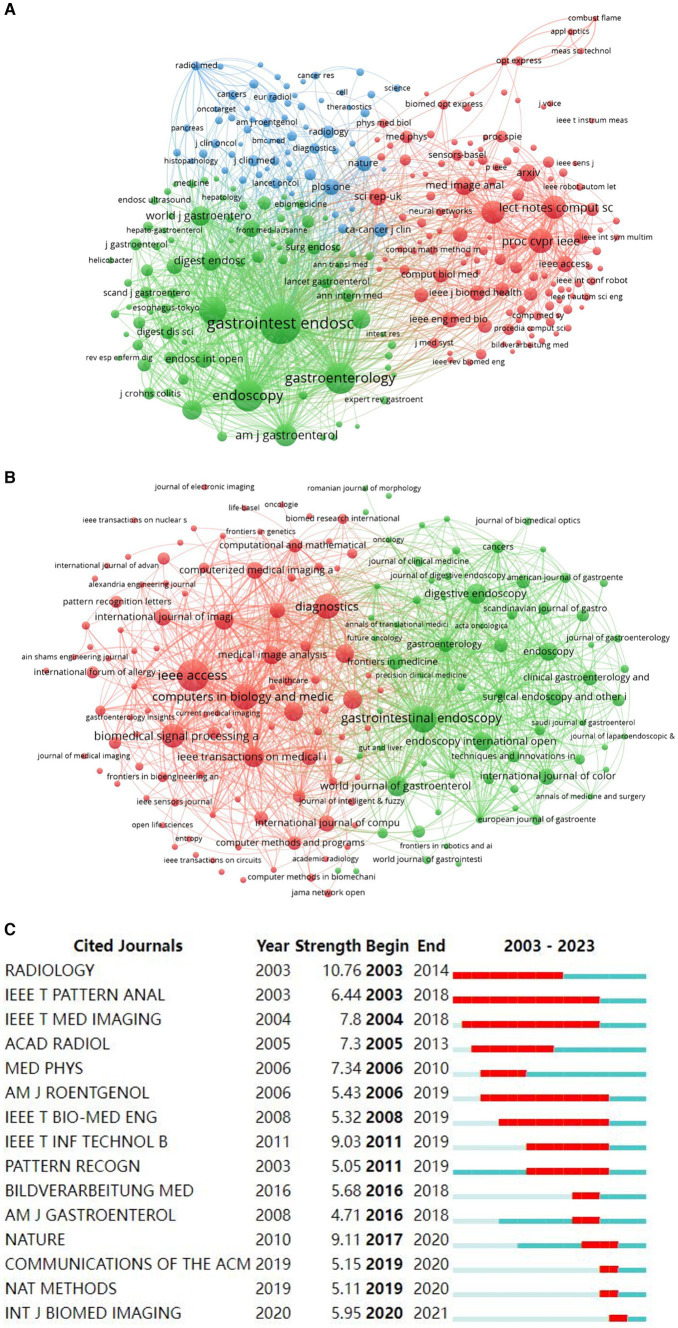
Literature screening flowchart.

Web of Science stands as a renowned database, esteemed for its expansive repository of high-calibre documents and potent search capabilities, rendering it the optimal selection for bibliometric scrutiny (24, 25). In the course of this investigation, the Web of Science Core Collection (WoSCC) (26) was harnessed to retrieve and procure all literature related to artificial intelligence (AI) in the domain of AI within gastrointestinal polyps and digestive endoscopy, spanning from January 1, 2003, to December 31, 2023. To secure a comprehensive review of the literature, prior studies were consulted, and a search strategy was devised for AI-linked gastrointestinal polyp research, encompassing the following terms: ((TS = (“artificial intelligence” OR “artificial neural network” OR “adversarial generative” OR “active learning” OR “Bayes network” OR “computational intelligence” OR “Convolutional Neural Networks” OR “Cellular Neural Network” OR “continual learning” OR “contrastive learning” OR “deep learning” OR “deep network” OR “deep neural network” OR “data mine” OR “data mining” OR “domain adaptation” OR “expert system” OR “feature extraction” OR “feature learning” OR “feature mining” OR “feature embedding” OR “few-shot learning” OR “feature selection” OR “graph learning” OR “graph mining” OR “intelligent learning” OR “instance segmentation” OR “image segmentation” OR “knowledge graph” OR “meta learning” OR “machine learning” OR “metric learning” OR “neural nets model” OR “neural network” OR “neural learning” OR “reinforcement learning” OR “Semantic segmentation” OR superpixel OR self-supervised OR “supervised learning” OR “semi-supervised” OR “transfer learning” OR “unsupervised learning” OR “unsupervised clustering”))) AND TS = (“Endoscop*” OR “Colonoscop*” OR “Gastroscop*” OR Digestive endoscop* OR Gastrointestinal Endoscop*) AND TS = (“polyp” OR “gastric polyps” OR “intestinal polyp”) AND DOP = (2003-01-01/2023-12-31). The identical approach was applied in devising the search strategy for AI research pertaining to digestive endoscopy, details of which are provided in the Supplementary material. Literature types excluded from consideration encompassed non-article and non-review, in addition to those authored in languages other than English. Two reviewers, ZiP and ZhP, were entrusted with the duty of evaluating the literature, employing the abstract and title as the basis for their assessment. Titles deemed irrelevant were independently eliminated during the initial screening process. In instances of divergence between the reviewers, consensus was reached through deliberation. The search and download of literature were concluded on January 1, 2024, within a single day. Figure 1 shows the literature inclusion process.

### 2.2 Data analysis and visualization

All the literature encompassed in this study possesses comprehensive records within the WoSCC. The Impact Factor (IF) for each journal was obtained by referring to the latest Journal Citation Reports (JCR), a pivotal metric for evaluating academic influence (27, 28).

**Figure 2:**
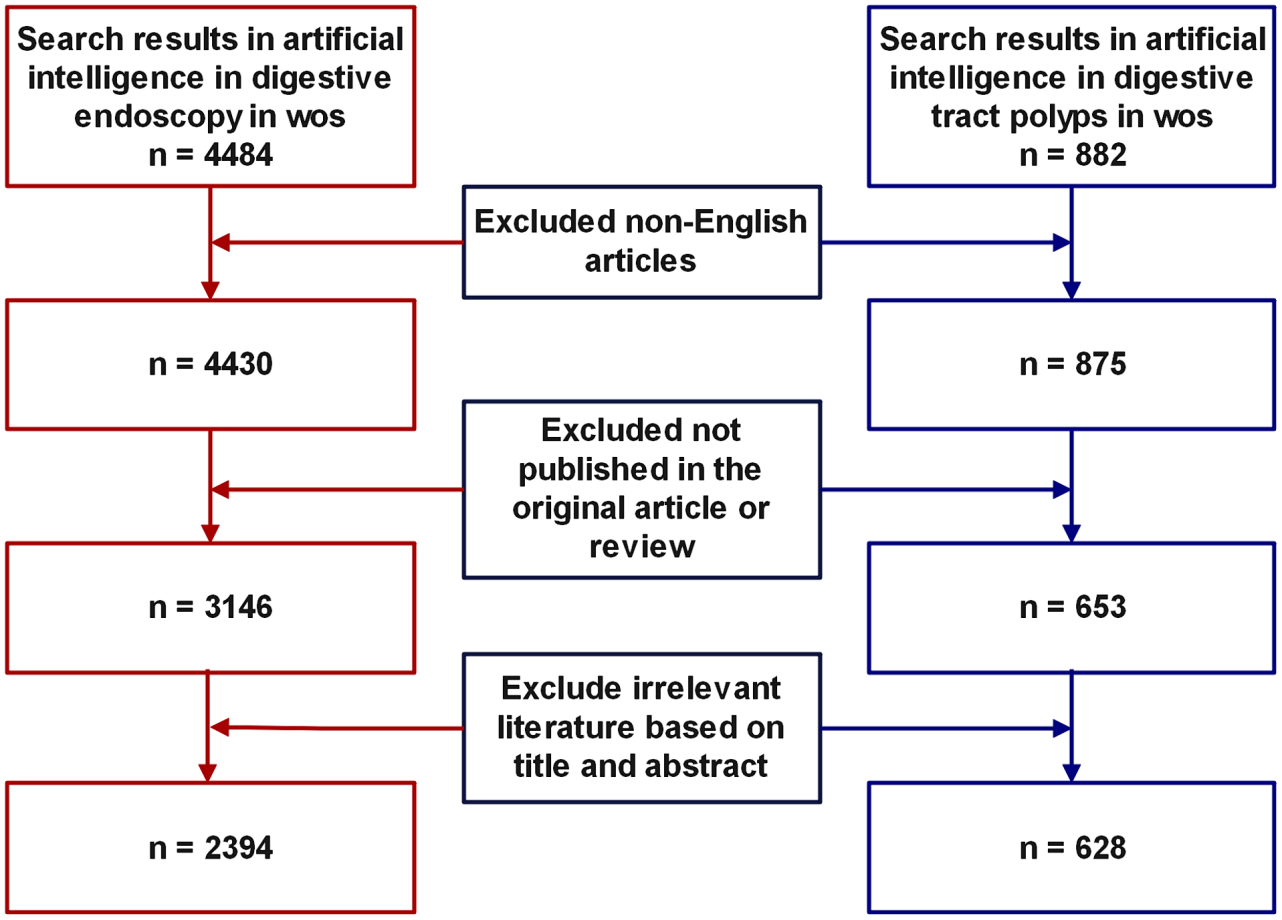
Global trends in publications and citations on AI in digestive endoscopy and AI for gastrointestinal polyps. **(A)** Annual number of published articles. **(B)** Annual cumulative number of articles. **(C)** Annual cumulative number of citations. **(D)** Annual *H*-index values of the publications. **(E)** Annual number of publications in the top 15 countries. **(F)** Trends in the proportion of AI for gastrointestinal polyps within the scope of AI in digestive endoscopy. (Ratio = AI for gastrointestinal polyps/AI in digestive endoscopy).

**Figure 3:**
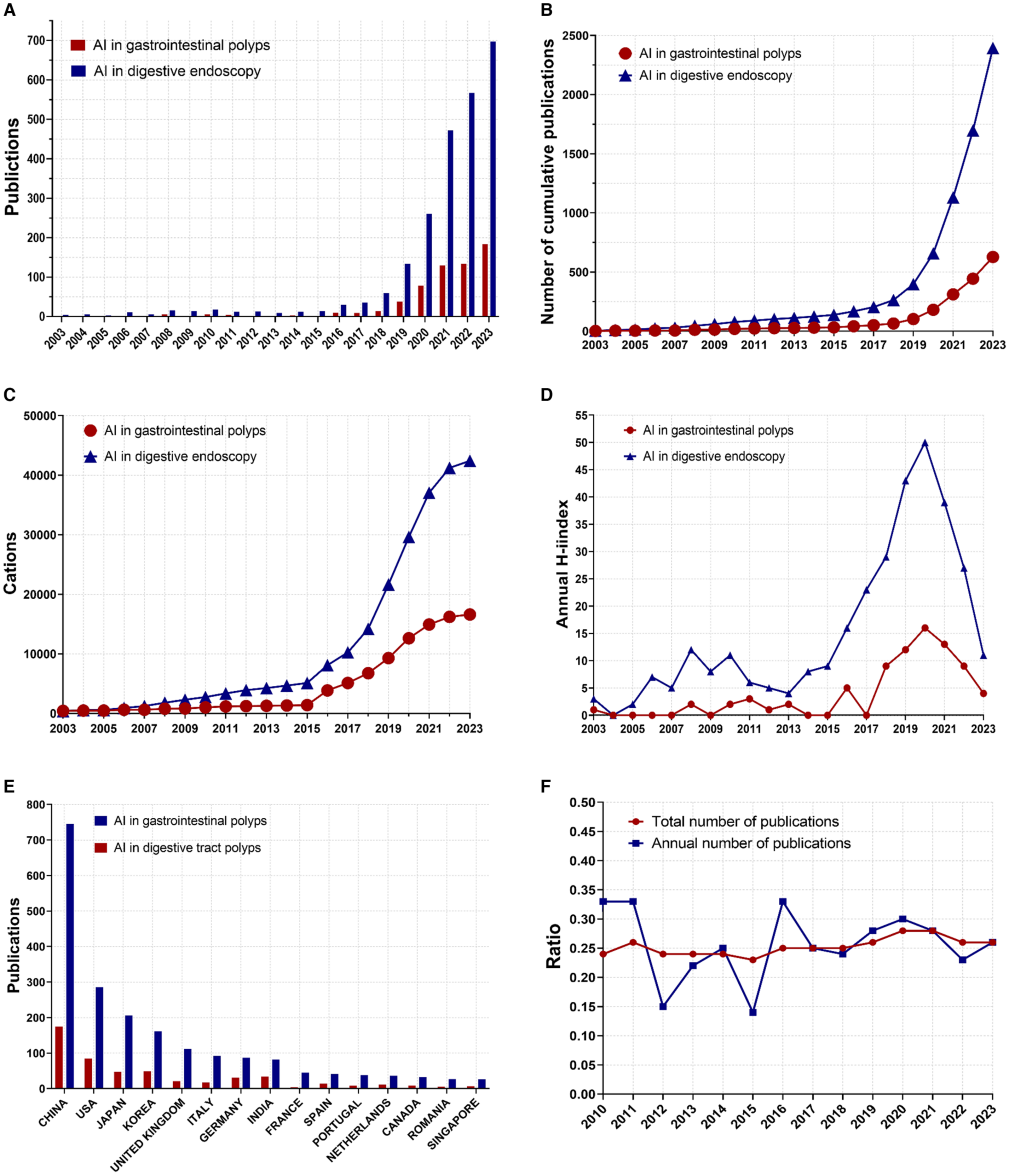
Author analysis in the field of AI for gastrointestinal polyps. **(A)** Author cooperation network. **(B)** The top 15 authors with the strongest citation bursts. **(C)** Productivity trends of the top authors over time.

**Figure 4:**
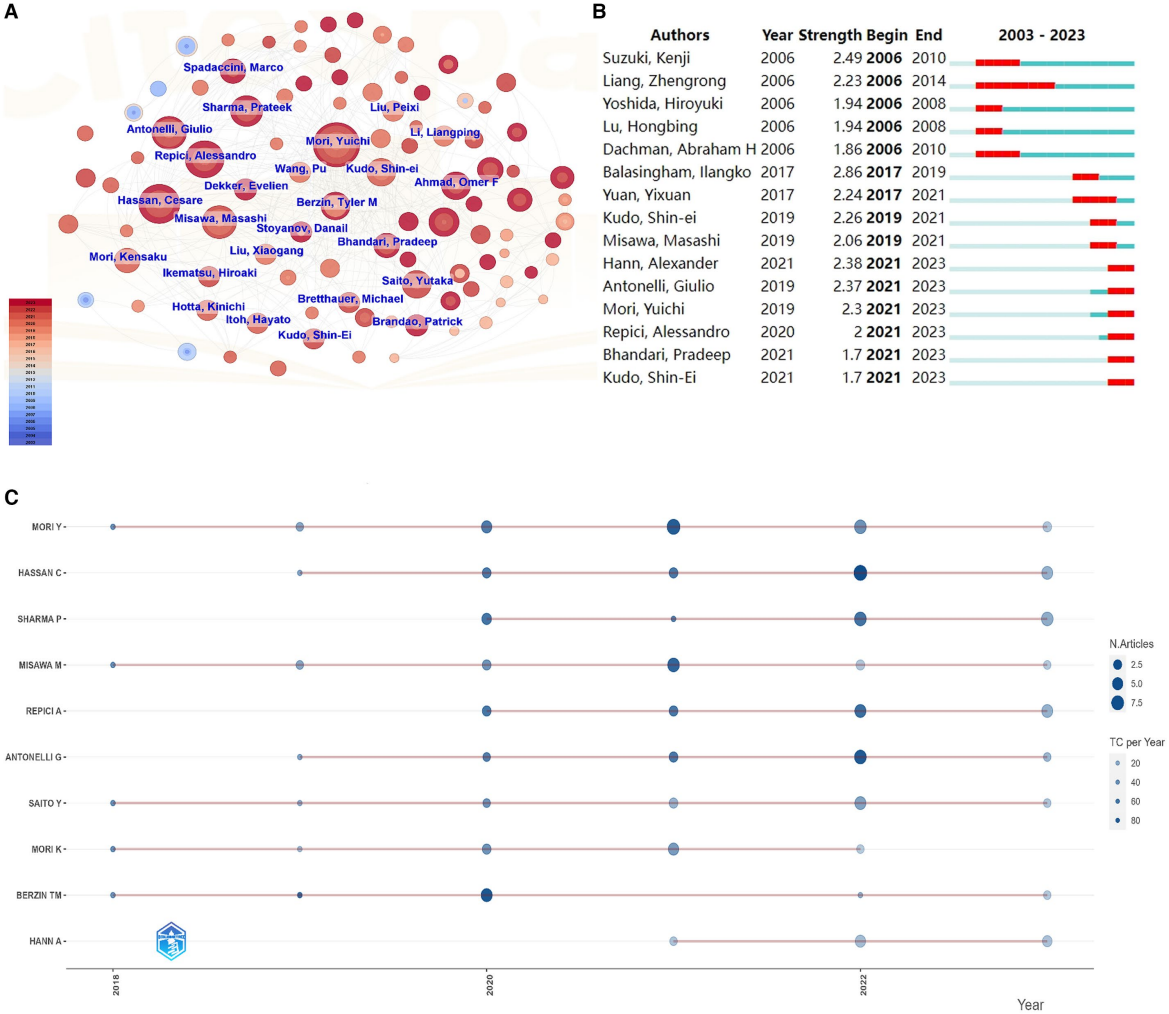
Keyword analysis in the field of AI for gastrointestinal polyps. **(A)** The top 15 keywords with the strongest citation bursts. **(B)** Timeline diagram. **(C)** Topic trends in AI for gastrointestinal polyps.

In this investigation, the Biblioshiny R package,^1^ a web interface for the Bibliometrix R package, was utilized for data analysis and visualization. This toolkit enabled preliminary analysis and provided visual representations of the data. The data presented in Figure 2 was sourced from the data module of the Bibliometrix R package, which features an interactive data export interface. After downloading the data, it was exported to GraphPad Prism 9.5.1 (29) for visualization. In addition, the Bibliometrix R package offers robust analytical capabilities and were utilized to analyze author data (Figure 3C) and keyword trends (Figure 4C). The size of the circles in the figure represents the frequency of keyword appearances, while the length of the blue lines indicates how long each keyword continues to receive attention.

**Figure 5:**
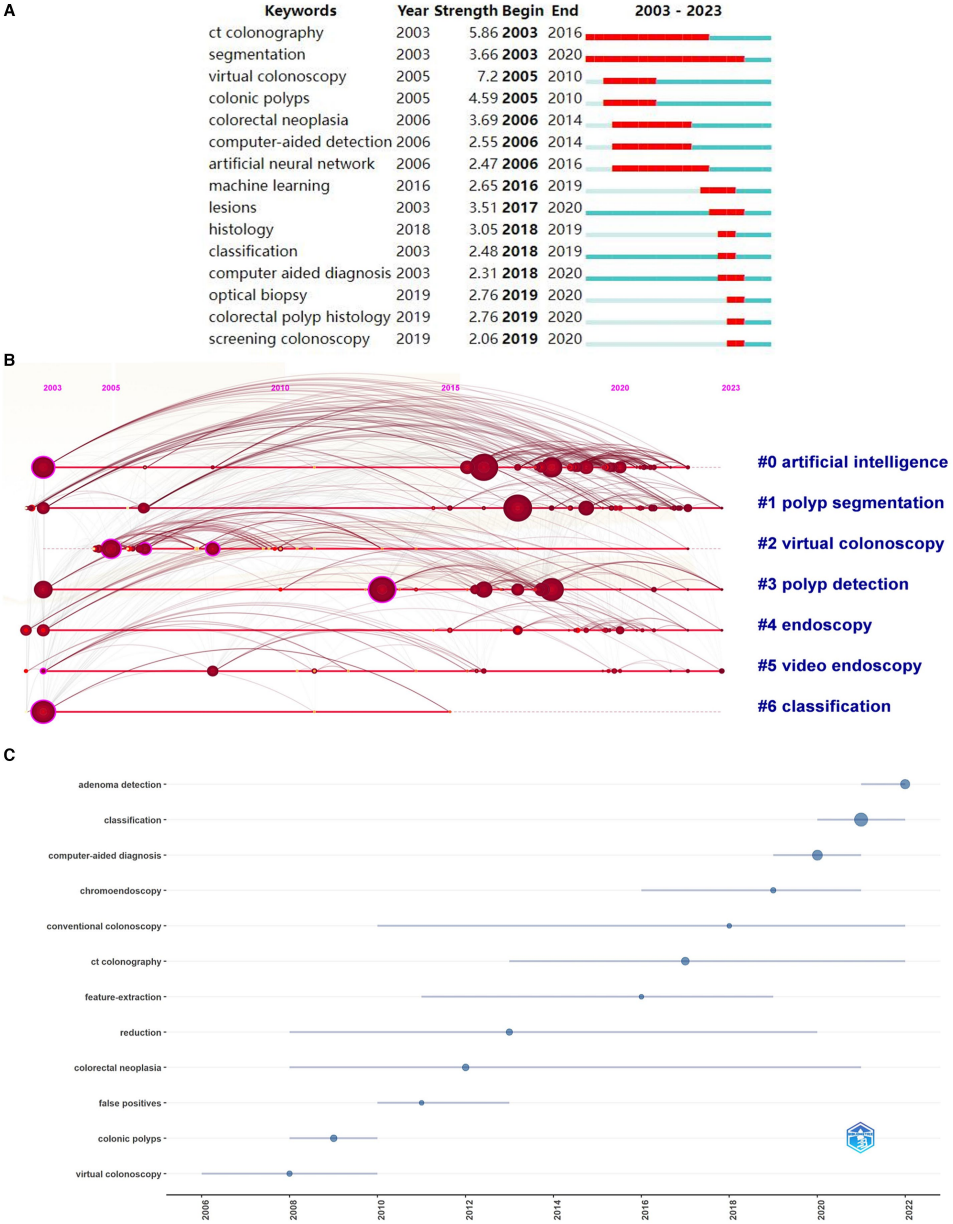
Publications and collaborations among different countries/regions and institutions. **(A)** Geographic distribution by countries/regions. **(B)** Chord diagram of publications and collaborations by country/region. **(C)** Chart of institutional cooperation. **(D)** The top 15 institutions with the strongest citation bursts.

**Figure 6:**
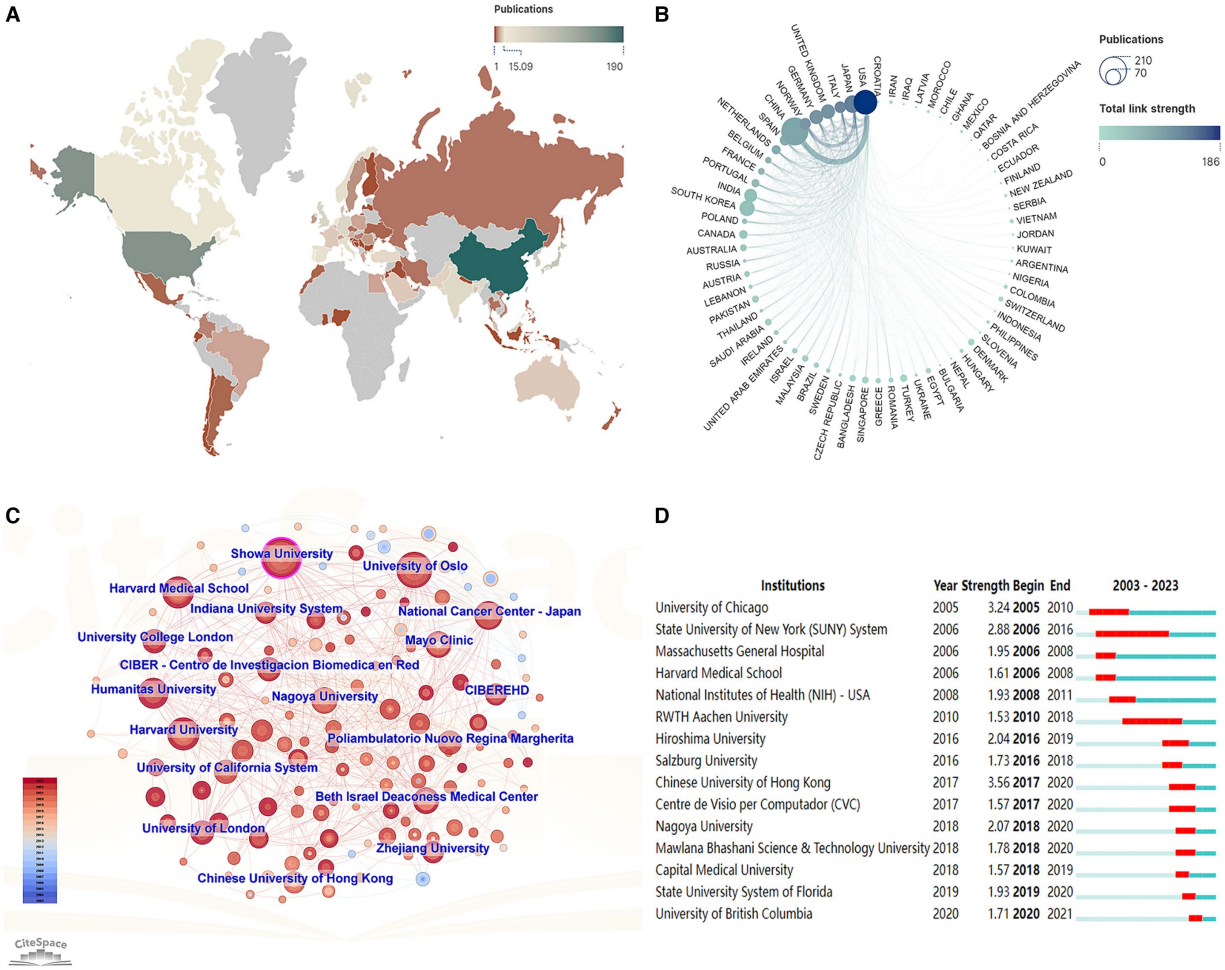
Analysis of journal contributions in the field of AI in digestive endoscopy and AI for gastrointestinal polyps. **(A)** Network of co-cited journals in the field of AI in digestive endoscopy. **(B)** Network of co-cited journals in the field of AI for gastrointestinal polyps. **(C)** The top 15 institutions with the strongest citation bursts in gastrointestinal polyps.

VOSviewer 1.6.19 (30) were called upon to analyse various facets, including countries, authors, institutions, journals, keywords, references, and citations. Additionally, SCImago Graphica 1.0.36 (31) was enlisted to scrutinise research outcomes and collaborations among diverse countries, culminating in the generation of a graphical representation of the findings. The geographical distribution map and chord diagram (Figures 5A,B) were created by first exporting national publication and collaboration data from VOSviewer and then visualizing it using the geographic mapping template in SCImago. Lastly, journal clustering diagrams (Figures 6A,B) were produced using the clustering module in VOSviewer.

CiteSpace 6.3.1 (32) is a widely used bibliometric tool that analyzes research trends, collaborations, and emerging topics across various fields over time. If certain institutions are concentrated within a specific period, we can refer to them as “burst institutions.” Burst words can indicate various stages of development within a field (33). The burst maps (Figures 3B, 4A, 5D, 6C) were generated using the “Burstness” module in CiteSpace software. CiteSpace also facilitated the visualization of collaboration networks (Figures 3A, 5C). For the keyword timeline diagram (Figure 4B), the clustering function in CiteSpace was employed to group all keywords, followed by visualization through the “Timeline View” module. In Figure 4B, the circles along each clustering result timeline represent the keywords associated with each category. The size of each circle reflects the frequency of the keyword’s occurrence, with larger circles indicating higher frequencies. The position of the circles along the timeline corresponds to the time of occurrence, where circles positioned further to the right indicating more recent keywords.

## 3 Result

### 3.1 General description of the retrieved publications

We conducted an extensive search for articles pertaining to the application of artificial intelligence (AI) in gastrointestinal polyps from 2003 to 2023, resulting in the identification of 628 articles. These articles encompassed contributions from 2,984 authors, represented 1,073 research institutions, and were published across 217 journals hailing from 66 countries or regions. Moreover, we located 2,394 publications on the application of AI in digestive endoscopy, featuring the involvement of 10,331 authors, 3,284 institutions, and a distribution across 579 journals and 74 countries or regions. A visual representation of the document selection process can be found in Figure 1, while supplementary search details are available in the Supplementary material.

The publication landscape of AI-related research in gastrointestinal polyps and digestive endoscopy from 2003 to 2023 is illustrated in Figure 2A. Notably, the trajectory of publications in both domains exhibits a shared pattern: stability characterised the period prior to 2016, after which the annual number of publications began to increase. Figure 2B provides insight into the cumulative annual publication figures for these fields. Preceding 2017, the rate of publication growth was relatively modest. However, over the ensuing 5 years (2017–2023), the number of publications within the AI gastrointestinal polyps field increased by approximately 12-fold (from 51 to 628), while the number of AI-related publications in digestive endoscopy increased by nearly tenfold (rising from 204 to 2,394). The annual citation index, as depicted in Figure 2C, reveals that the count of citations for AI in the realm of digestive endoscopy exhibited a gradual increase starting in 2007, with a marked acceleration post-2015, reaching a zenith of 42,409 citations. The annual *H*-index for publications within both domains, as presented in Figure 2D, experienced a pronounced upsurge after 2015, culminating in a peak in 2020. Figure 2E offers a depiction of the number of publications within the top 15 countries by publication count in both fields, with China leading the list. Figure 2F outlines the annual number of publications and the cumulative number of publications in both fields (AI in gastrointestinal polyps/AI in digestive endoscopy). Though the proportion of published papers fluctuates from year to year, the proportion of cumulative published papers has generally stabilized above 0.25 after 2019.

### 3.2 Analysis of countries and institutions

Within the domains of AI-assisted gastrointestinal polyp diagnosis and digestive endoscopy, China, the USA, Korea, and Japan occupy the top four positions in terms of publication volume. Notably, Norway boasts an impressive average of 58.90 citations per article within the realm of AI gastrointestinal polyps (Table 1). A total of 1,073 institutions were actively engaged in AI-related research concerning gastrointestinal polyps, while 3,284 institutions participated in AI-related research pertaining to digestive endoscopy. The number and distribution of these institutions serve as a reflection of the global landscape and trends in AI applications within gastroenterology. Harvard University emerges as the leading institution in both fields (Table 2).

**Table 1:** Top 10 productive countries/regions related to AI digestive endoscopy and AI gastrointestinal polyps.

AI in digestive endoscopy				AI in gastrointestinal polyps			
Country	*N*	Citation	Average citation	Country	*N*	Citation	Average citation
China	745	10,701	14.40	China	175	3,761	21.50
USA	286	8,826	30.90	USA	84	4,093	48.70
Japan	206	4,993	24.20	Korea	49	958	19.60
Korea	162	2,300	14.20	Japan	47	1,626	34.60
United Kingdom	112	2,065	18.40	Germany	31	397	12.80
Italy	92	1,741	18.90	India	34	146	4.30
Germany	87	1,333	15.30	United Kingdom	21	432	20.60
India	82	466	5.70	Italy	17	721	42.40
France	45	1,374	30.50	Spain	14	210	15.00
Spain	41	602	14.70	Norway	11	648	58.90

**Table 2:** Top 10 productive institutions related to AI digestive endoscopy and AI gastrointestinal polyps.

AI in digestive endoscopy				AI in gastrointestinal polyps			
Institution	Country	*N*	Citation	Institution	Country	*N*	Citation
Wuhan University	China	156	1,092	Harvard University	USA	40	698
University of London	USA	150	101	University of Oslo	Norway	39	1,275
Harvard University	USA	99	3,056	Showa University	Japan	33	1,269
University of Oslo	China	73	1,674	National Cancer Institute—Japan	Japan	28	830
Universidade do Porto	Portugal	69	260	University College London	USA	24	449
Chinese University of Hong Kong	China	68	1,960	State University of New York	USA	23	364
Chinese Academy of Science	China	66	741	Harvard Medical School	USA	21	1,441
Zhejiang University	China	62	601	Seoul National University	Korea	21	105
Helmholtz Association	Germany	58	382	Veterans Health Administration (VHA)	USA	20	273
Showa University	Japan	58	1,856	University of California System	USA	19	92

The distribution of publications pertaining to AI in the field of gastrointestinal polyps and the collaborations among countries and institutions are graphically represented in Figure 5. A global perspective reveals that the majority of papers originated from Western Europe, North America, and East Asia (Figure 5A). To gauge the strength of inter-country connections within the collaboration network, total link strength (TLS) was utilized. The top three countries with the highest TLS are the United States (186), the Japan (133), and Italy (127), attesting to their extensive and robust collaborative efforts with other nations. China, the foremost contributor of publications, maintains close partnerships with the United States, South Korea, and Japan, while collaboration among other developing nations remains relatively modest (Figure 5B). The network of collaboration among institutions is depicted in Figure 5C. In general, the collaborative relationships among these institutions appear somewhat distant, with the majority of collaborations being concentrated in institutions boasting a substantial number of publications. The Chinese University of Hong Kong exhibits the highest burst intensity (3.56), indicating that this institution had the highest research activity during the outbreak period (2017–2020). Additionally, the University of British Columbia, a new and emerging institution, has witnessed a growing trend since 2020 (Figure 5D).

### 3.3 Analysis of authors

Figure 3A portrays the author collaboration network diagram concerning AI-related gastrointestinal polyp research. This network illustrates that collaborations between authors are more frequent and robust, particularly amongst authors with a substantial volume of publications, as evident from the size of the nodes. This observation suggests that highly productive authors tend to establish stable and influential research groups or clusters within this domain. Notably, Yuichi Mori (26), Cesare Hassan (21), and Prateek Sharma (18) emerge as the top three authors in terms of publication quantity, maintaining more frequent collaborations with other authors (Table 3). Among these authors, Ilangko Balasingham boasts the highest burst intensity over the past two decades (2.86), primarily due to a widely-cited paper on wireless capsule endoscopy published in 2018 (34). Moreover, the post-2021 period has witnessed six scholars exhibiting an explosive state (Figure 3B), indicative of their rising influence and impact within the field. Figure 3C provides an overview of the top authors’ publications within the past 5 years, where the majority of authors have consistently published throughout this period. However, Repici A. and Sharma P. displayed a more significant contribution during the latter half of this timeframe.

**Table 3:** Top 10 productive authors related to AI digestive endoscopy and AI gastrointestinal polyps.

AI in digestive endoscopy					AI in gastrointestinal polyps				
Author	Country	*N*	Citation	Average citation	Author	Country	*N*	Citation	Average citation
Yuichi Mori	Japan	52	1,613	31.02	Yuichi Mori	Japan	27	1,179	43.67
Honggang Yu	China	46	840	18.26	Cesare Hassan	Italy	22	994	45.18
Cesare Hassan	Italy	42	1,106	26.33	Prateek Sharma	USA	19	750	39.47
Prateek Sharma	USA	40	986	24.50	Repici Alessandro	USA	18	774	43.00
Repici Alessandro	USA	37	898	24.27	Misawa Masashi	Japan	18	951	52.83
Misawa Masashi	Japan	36	1,405	39.03	Yutaka Saito	Japan	15	777	51.80
Tomohiro Tada	Japan	35	1,953	55.80	Antonelli Giulio	Italy	15	947	63.13
Lianlina Wu	China	32	548	17.13	Kensaku Mori	Japan	12	706	58.83
Kensaku Mori	Japan	29	1,176	40.55	Alexander Hann	Germany	11	71	6.45
Jun Liu	China	28	564	9.14	Wang Pu	China	11	1,138	103.45

### 3.4 Analysis of journals

A total of 579 journals have disseminated articles within the realm of AI digestive endoscopy, with VOSviewer facilitating cluster analysis to categorise them. The results, as depicted in Figure 6A, group the journals into three clusters: endoscopy journals (Gastroenterology, Endoscopy, and Digestive Endoscopy), artificial intelligence journals, and medical journals (e.g., Computers in Biology and Medicine) encapsulated within the intermediate blue cluster node. In the arena of AI gastrointestinal polyps, there are 217 journals, and the clustering outcomes primarily pertain to endoscopy and artificial intelligence (Figure 6B). This implies a concentration of AI gastrointestinal polyp research on the development of AI techniques for endoscopy, encompassing aspects like polyp detection, characterisation, and resection. In reference to the top 15 cited journals with the most substantial citation bursts in both fields, Radiology (10.76) exhibits the highest burst intensity, closely followed by Nature (9.11) (Figure 6C). Among the top 10 journals in AI digestive endoscopy and AI gastrointestinal polyps with the greatest number of publications, IEEE Access, Diagnostics, and Gastrointestinal Endoscopy stand out (Table 4).

**Table 4:** Top 10 most productive journals related to AI digestive endoscopy and AI gastrointestinal polyps.

Journal	*N*	Citation	Average citation	IF (2023)	JCR (2023)	Journal	*N*	Citation	Average citation	IF (2023)	JCR (2023)
Gastrointestinal Endoscopy	85	3,533	41.56	7.70	Q1	IEEE Access	26	521	20.04	3.90	Q2
Diagnostics	78	369	4.73	3.60	Q3	Diagnostics	24	126	5.25	3.60	Q3
IEEE Access	68	990	14.56	3.90	Q2	Gastrointestinal Endoscopy	18	1,063	59.06	7.70	Q1
Scientific Reports	62	952	15.35	4.60	Q2	Computers in Biology and Medicine	17	406	23.88	7.70	Q1
Computers in Biology and Medicine	55	1,165	21.18	7.70	Q1	Digestive Endoscopy	17	291	17.12	5.30	Q2
Digestive Endoscopy	50	926	18.52	5.30	Q2	World Journal of Gastroenterology	16	247	15.44	4.30	Q2
World Journal of Gastroenterology	48	706	14.71	4.30	Q2	Biomedical Signal Processing and Control	14	142	10.14	5.10	Q2
International Journal of Computer Assisted Radiology and Surgery	42	547	10.88	3.00	Q3	Endoscopy	12	557	46.42	9.30	Q1
Endoscopy	39	1,426	36.56	9.30	Q1	IEEE Transactions on Medical Imaging	12	2,683	223.58	8.52	Q1
Biomedical Signal Processing and Control	14	328	23.43	5.10	Q2	Endoscopy International Open	11	118	10.72	/	/

### 3.5 Analysis of keywords

Among the 628 studies pertaining to AI in gastrointestinal polyps, a comprehensive examination of 1,637 keywords was carried out using cluster analysis through CiteSpace. The outcomes of this clustering endeavour were categorised into 7 distinct clusters, denoted as: “artificial intelligence” (#0), “polyp segmentation” (#1), “virtual colonoscopy” (#2), “polyp detection” (#3), “endoscopy” (#4), “video endoscopy” (#5), and “classification” (#6). A detailed inspection of these keyword categories unveiled noteworthy temporal patterns. Notably, three categories, namely “artificial intelligence” (#0), “polyp segmentation” (#1), and “polyp detection” (#3), exhibited the emergence of a substantial proportion of new keywords post-2010. Conversely, two categories, “virtual colonoscopy” (#2) and “classification” (#6), primarily featured new keywords prior to 2010. Notably, the growth in “artificial intelligence” (#0), “polyp segmentation” (#1), and “polyp detection” (#3) in 2010 surpassed the expansion of other clusters (Figure 4B) It is worth highlighting that the analysis of keyword bursts plays a pivotal role in discerning evolving research trends within an academic domain (35). In light of this, a citation analysis was performed on the keywords within this field, revealing a notable reduction in the duration of keyword outbreaks post-2010. Among the keywords exhibiting the highest outbreak intensity, “CT colonography” (5.86) was initially introduced in 2003 and continued to experience bursts of citations until 2016. Conversely, the term “computer-aided diagnosis” was first introduced in 2003 but garnered substantial attention in the literature by 2018, marked by several citation bursts. In tandem, related terms such as “optical biopsy,” “colorectal polyp histology,” and “screening colonoscopy” also witnessed heightened interest during this period (Figure 4A). Conversely, Figure 4C spotlights the most recent trending topics, with “adenoma detection,” “artificial intelligence,” and “classification” standing out. These terms reflect the growing adoption of deep learning technology within colonoscopy, a development pioneered by Bulluck and Hausenloy (36) in their seminal paper on two types of computer-aided diagnosis, published in Nature Medicine in 2018.

**Figure 7:**
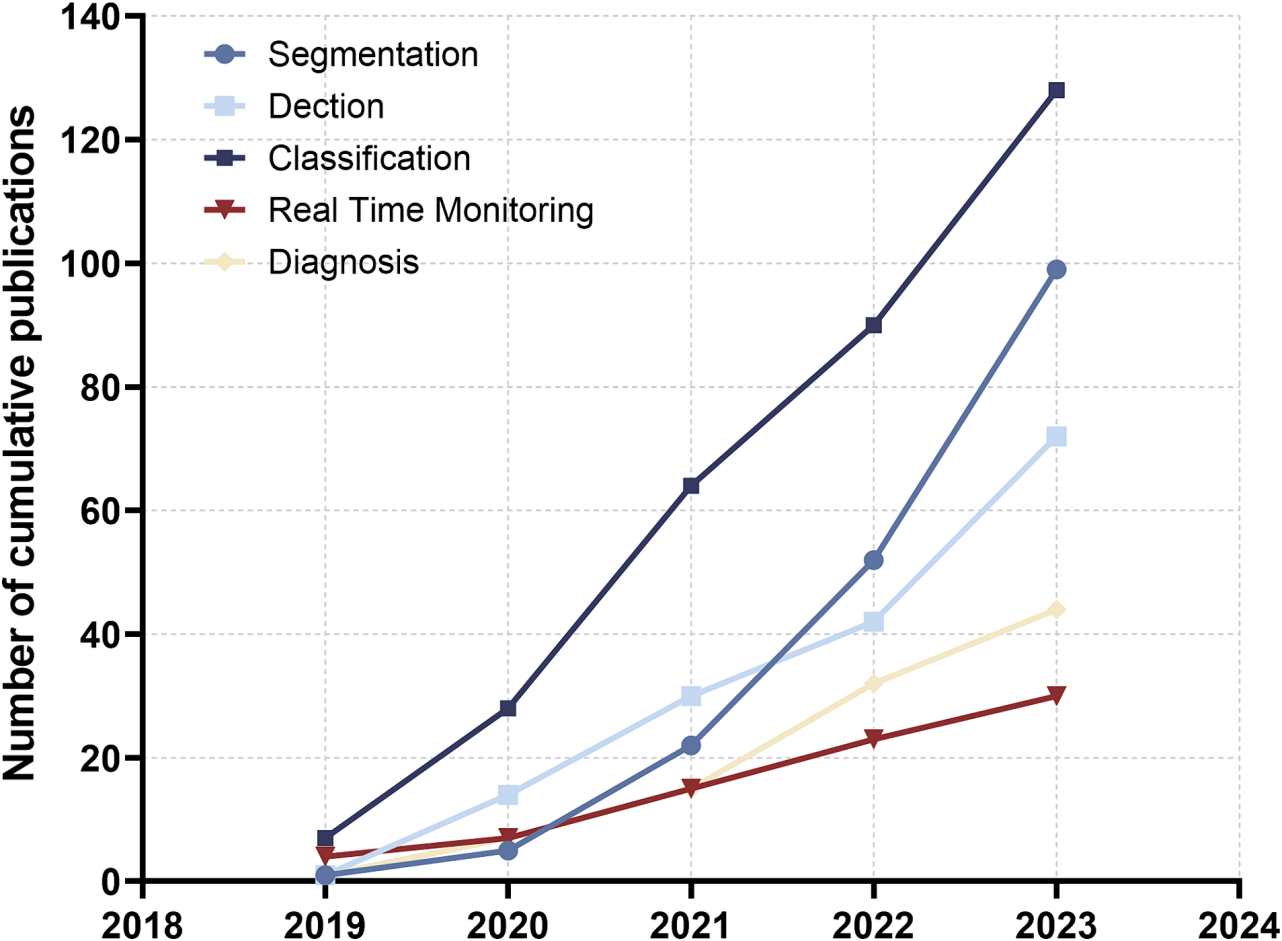
The number of cumulative publications on AI applications in digestive tract polyp segmentation, detection, classification, real-time monitoring and AI-assisted diagnosis in the past 5 years.

Aligning with the practical clinical context of AI, it is apparent that the prevailing topics in AI deep learning-assisted colonoscopy polyp diagnosis can be broadly categorised into three domains: polyp detection, classification, and segmentation (37, 38). In recent years, these three technologies have found gradual application in practical clinical application, culminating in the development of a real-time detection system and an AI-assisted polyp diagnosis system (39). The prevalence of publications in the field of AI-assisted polyp classification surpasses that of AI-assisted polyp segmentation, with the latter exhibiting the second highest number of publications. Notably, research on segmentation demonstrates the most rapid growth rate among these areas. Furthermore, a discernible increase in publications pertaining to real-time polyp surveillance and diagnosis emerged around the year 2020 (Figure 7). This trend underscores the progressive integration of AI technologies into the realm of polyp diagnosis, signifying a phase of substantial technological advancement and application within the field.

### 3.6 Analysis of the most cited references

A total of 628 AI-related studies on gastrointestinal polyps were identified, spanning the publication period from 2003 to 2023. Within this corpus, 79 studies garnered more than 50 citations. Table 5 provides insight into the top 10 cited studies pertaining to AI in gastrointestinal polyps. These studies predominantly found their place within high-impact journals, including GUT, Gastroenterology, Nature Biomedical Engineering, and IEEE Transactions on Medical Imaging, where each has accumulated over 250 citations. It is noteworthy that seven of these studies were released within the last 5 years, underscoring the recent surge of interest and growth in this research domain.

**Table 5:** Top 10 most cited articles in the field of AI gastrointestinal polyps from 2003–2023.

Title	Journal	Author	Year	Citation
Convolutional neural networks for medical image analysis: full training or fine tuning?	IEEE Transactions on Medical Imaging	Tajbakhsh N. et al.	2016	349
Real-time automatic detection system increases colonoscopic polyp and adenoma detection rates: a prospective randomised controlled study	GUT	Wang P. et al.	2019	422
Deep learning localizes and identifies polyps in real time with 96% accuracy in screening colonoscopy	Gastroenterology	Urban G. et al.	2018	391
Real-time differentiation of adenomatous and hyperplastic diminutive colorectal polyps during analysis of unaltered videos of standard colonoscopy using a deep learning model	GUT	Byrne M. F. et al.	2019	366
Automated polyp detection in colonoscopy videos using shape and context information	IEEE Transactions on Medical Imaging	Tajbakhsh N. et al.	2016	339
Computer-aided tumor detection in endoscopic video using color wavelet features	IEEE Transactions on Information Technology in Biomedicine	Karkanis S. A. et al.	2003	333
Real-time use of artificial intelligence in identification of diminutive polyps during colonoscopy	Annals of internal Medicine	Mori Y. et al.	2018	295
Development and validation of a deep-learning algorithm for the detection of polyps during colonoscopy	Nature Biomedical Engineering	Wang P. et al.	2018	269
Efficacy of real-time computer-aided detection of colorectal neoplasia in a randomized trial	Gastroenterology	Repici A. et al.	2020	266
Accurate classification of diminutive colorectal polyps using computer-aided analysis	Gastroenterology	Chen P. J. et al.	2018	253

Within the realm of AI in digestive endoscopy, a grand total of 2,394 pertinent literature pieces were encompassed, with 159 of them amassing over 50 citations. The top 10 most-cited articles were notably affiliated with three journals: Gastroenterology, GUT, and IEEE Transactions on Medical Imaging (Supplementary Table S1). Remarkably, six of the top 10 most-cited studies in AI digestive endoscopy also featured within the top 10 most-cited studies in AI gastrointestinal polyps, as delineated in Table 5. This signifies that AI-related research concerning gastrointestinal polyps is an advanced and well-established theme in the domain of AI applications in digestive endoscopy. It has not only garnered substantial attention but also yielded significant advancements aimed at enhancing the quality and precision of colonoscopy.

## 4 Discussion

This study represents the first integration of AI in digestive endoscopy, presenting a comprehensive analysis of the evolving trends in AI within the domain of gastrointestinal polyps over the past two decades through bibliometric techniques. Our investigation not only reviews the trajectory of AI-assisted research on gastrointestinal polyps but also provides a comparative analysis, shedding light on the proportion and research emphasis of AI in gastrointestinal polyp recognition when juxtaposed with the broader context of AI research in digestive endoscopy.

Gastrointestinal polyps, recognised as the precursors to numerous digestive tumours, have garnered considerable research interest, particularly pertaining to early detection. The rapid advancement of artificial intelligence (AI) technology in both the sphere of digestive endoscopy and gastrointestinal polyps since 2017 is evident, with the former displaying a more accelerated growth rate (32, 40, 41). The quantity of publications and the annual H-index in both domains have also exhibited a notable upswing since 2017. Furthermore, from 2010 to 2023, there has been a stabilization observed in the number of AI-related publications within the realm of digestive polyps, constituting approximately a quarter (25%) of the broader field of AI digestive endoscopy.

In our exploration of the prominent nations and institutions in the domain of AI gastrointestinal polyps, it becomes apparent that China, The USA, and Japan have emerged as the most significant contributors. China, in particular, commands the highest number of publications and citations in both AI digestive endoscopy and gastrointestinal polyps, spearheading the global collaborative network. Among the research institutions operating within these two fields, Harvard University, Showa University and University of Oslo all lead in the number of publications, occupying a central role within the institutional cooperative network. Yuichi Mori, a pivotal figure in the AI gastrointestinal polyps field, has significantly impacted this domain. His foray into this field began in 2018 when he and his colleagues employed an AI model to forecast preoperative lymph node metastasis in colorectal cancer, facilitating decisions regarding the necessity of additional surgery post-resection of T1 colorectal cancer. This seminal publication marked his debut in the AI gastrointestinal polyps arena, after which he continued to play an active role. In 2022, Mori et al. (42) conducted an evaluation of AI polyp detection methods, offering insights and advising endoscopists to exercise increased vigilance during the procedure.

The keyword clustering results pertaining to AI gastrointestinal polyps encompass five categories, all closely tied to computer AI algorithms. The keyword timeline chart unveils that the category represented by #2, virtual colonoscopy, primarily flourished between 2006 and 2009, while categories #0, artificial intelligence, #1, polyp segmentation, and #3, polyp detection, have witnessed the emergence of new terminology in recent years. Of the 10 most cited studies on AI gastrointestinal polyps, a notable eight have been published within the past 5 years. This observation underscores the substantial strides made in the application of AI technology within the gastrointestinal polyps domain over this five-year span. Regarding polyp detection, Karkanis S. A. et al. introduced a feature extraction method for tumour detection grounded in a colour feature scheme in 2003, a study that garnered 314 citations, securing the fourth position within the field of AI gastrointestinal polyps. Subsequently, progress in polyp detection was hampered by limitations in computing power and AI technology until 2016 (43, 44), when a significant breakthrough materialised. Tajbakhsh N. et al. devised a polyp detection method premised on the extraction of polyp shape features through a hybrid context shape approach. In 2019, Wang et al. (45) advanced an automatic detection system for polyps and adenomas, conducting a prospective randomized controlled trial, which demonstrated the system’s capacity to enhance the adenoma detection rate. This study, published in the GUT journal, earned the top position in AI gastrointestinal polyps based on citations (349). Additionally, we observe that AI-assisted real-time monitoring of polyps has also demonstrated impressive progress in the last 5 years. Beyond Wang’s P. et al. research in 2019, Mori et al. (46) executed a prospective study in 2018 to evaluate their team’s computer-aided diagnosis system for small polyps. This system relies on the deep learning-based endoscopic image detection algorithm pioneered by their team in 2016. Furthermore, in 2019, Byrne et al. (47) harnessed a DCNN network model for real-time analysis of adenomas or hyperplastic polymers. An in-depth review of the literature readily reveals that, after years of technological accumulation, the last 5 years have borne witness to the emergence of commendable detection systems within the field of real-time polyp detection.

In recent years, bibliometric analysis has gained prominence for scrutinizing the research trends concerning AI within the domain of digestive endoscopy. This analytical approach aims to appraise the accuracy of AI in addressing diverse digestive tract maladies (48, 49). The current study expands this investigative purview by conducting a nuanced exploration of the shifts in AI research focused on digestive polyps. This analysis includes a comparative dimension with the broader sphere of AI digestive endoscopy, emphasizing the pivotal role of AI in the latter.

Currently, the theoretical accuracy of artificial intelligence (AI) algorithms can exceed 95% (50, 51). However, in practical applications within the field of AI-assisted gastrointestinal polyp detection, the accuracy is not as high. In 2022, Minegishi et al. (52) published a study in *Gastroenterology* on an AI-assisted real-time diagnostic system for colorectal lesions. Their system demonstrated a sensitivity of 95.8% for small tumors, but the specificity was only 67.0%, indicating a relatively high rate of false positives in clinical applications. This discrepancy highlights that the accuracy of real-time diagnostic systems in clinical practice has not yet reached the theoretical levels.

Despite this, significant advancements have been made in AI real-time diagnostic systems over the past 5 years. As shown in Figure 7, the number of publications on AI real-time detection and diagnostic systems in the gastrointestinal field was less than 10 in 2019, but by 2023, this number had increased approximately fivefold. Moreover, the accuracy of AI systems in clinical applications has also improved substantially over the past 5 years. In 2019, real-time systems were in the early stages of realizing the practical application of AI in gastroenterology, with less than ideal accuracy. However, in the last 3 years, the accuracy of systems in clinical applications has approached 80%, although the accuracy for certain specific lesions remains suboptimal (51, 52). Due to the current limitations in the accuracy of real-time detection systems and their lower accuracy for diverse lesions, the clinical application of AI real-time detection systems is still limited to small population groups (53).

The data from this study indicate that AI technology only began to assist in the segmentation, classification, and detection of gastrointestinal polyps in 2019, with AI-assisted real-time diagnosis systems emerging within the past 5 years. Currently, the accuracy and scope of AI technology applications in gastroenterology require further improvement. However, the continuous development of AI technology has led to a steady increase in both the quantity and quality of research on AI-assisted real-time diagnosis systems for gastrointestinal diseases. In the future, the improvement of the accuracy of AI real-time diagnosis systems and the promotion of clinical applications may be promising.

## 5 Limitations

This study exhibits certain limitations with regard to its literature selection criteria. Firstly, it exclusively encompasses English-language literature, potentially leading to the exclusion of valuable non-English sources, thereby introducing a measure of bias. Secondly, the study restricts its consideration to articles and reviews as the sole valid literary formats, thereby omitting other potentially relevant formats, such as conference papers, books, and letters. Thirdly, it confines its scope to literature published from 2003 onwards, which may inadvertently disregard some earlier pioneering works. Finally, our exclusive reliance on the Web of Science Core Collection as the sole source of literature may have resulted in the omission of relevant materials available in other databases.

## 6 Conclusion

With the rapid advancement of AI technology, its application in gastroenterology has extended beyond traditional AI-assisted tasks such as polyp segmentation, classification, and detection. AI is now being employed for real-time diagnosis and detection of gastroenterological diseases. The growing interest in AI within this field underscores its increasing importance. However, challenges remain regarding the accuracy and widespread adoption of AI-assisted real-time detection systems, suggesting that AI holds significant potential for further development in gastroenterology.

## Data Availability

Publicly available datasets were analyzed in this study. This data can be found at: the datasets generated and/or analyzed during the current study are available in the Science Citation Index Expanded (SCI-Expanded 1999-present) of Clarivate Analytics’ Web of Science Core Collection (WoSCC) repository, which can be accessed at https://www.webofscience.com/wos/alldb/basic-search.

## Footnotes

^1^https://bibliometrix.org/Biblioshiny.html
