# Supplementary material for: Comparative bibliometric analysis of artificial intelligence-assisted polyp diagnosis and AI-assisted digestive endoscopy: trends and growth in AI gastroenterology (2003–2023)
Source: Front Med (Lausanne). 2024 Sep 18;11:1438979. doi: 10.3389/fmed.2024.1438979 (PMC11445022; doi:10.3389/fmed.2024.1438979)
Supplement: Supplementary file 1 [file Data_Sheet_1.DOCX]

Supplementary Material

# Supplementary Data

The search strategy of AI digestive endoscopy:

((TS=(“artificial intelligence” OR “artificial neural network” OR “adversarial generative” OR “active learning” OR “Bayes network” OR “computational intelligence” OR “Convolutional Neural Networks” OR “Cellular Neural Network” OR “continual learning” OR “contrastive learning” OR “deep learning” OR “deep network” OR “deep neural network” OR “data mine” OR “data mining” OR “domain adaptation” OR “expert system” OR “feature extraction” OR “feature learning” OR “feature mining” OR “feature embedding” OR “few-shot learning” OR “feature selection” OR “graph learning” OR “graph mining” OR “intelligent learning” OR “instance segmentation” OR “image segmentation” OR “knowledge graph” OR “meta learning” OR “machine learning” OR “metric learning” OR “neural nets model” OR “neural network” OR “neural learning” OR “reinforcement learning” OR “Semantic segmentation” OR superpixel OR self-supervised OR “supervised learning” OR “semi-supervised” OR “transfer learning” OR “unsupervised learning” OR “unsupervised clustering”)) AND TS=(“Endoscop*” OR “Colonoscop*” OR “[Gastroscop](javascript:;)*” OR [Digestive endoscop*](javascript:;) OR [Gastrointestinal Endoscop*](javascript:;)) And DOP=(2003-01-01/2023-12-31)
