# Supplementary material for: Comparative bibliometric analysis of artificial intelligence-assisted polyp diagnosis and AI-assisted digestive endoscopy: trends and growth in AI gastroenterology (2003–2023)
Source: Front Med (Lausanne). 2024 Sep 18;11:1438979. doi: 10.3389/fmed.2024.1438979 (PMC11445022; doi:10.3389/fmed.2024.1438979)
Supplement: Supplementary file 2 [file Table_1.DOCX]

Supplementary Material

# Supplementary Tables

**Table S1.** Top 10 most cited articles in the field of AI digestive endoscopy from 2003-2023.

| **Title** | **Journal** | **Author** | **Year** | **Citation** | **AAS** |
| --- | --- | --- | --- | --- | --- |
| Application of artificial intelligence using a convolutional neural network for detecting gastric cancer in endoscopic images | *[Gastric Cancer](https://www.springer.com/journal/10120/)* | Hirsawa T; et al | 2018 | 355 | 28 |
| Real-time automatic detection system increases colonoscopic polyp and adenoma detection rates: a prospective randomised controlled study | *GUT* | Wang P; et al | 2019 | 349 | 594 |
| Deep Learning Localizes and Identifies Polyps in Real Time With 96% Accuracy in Screening Colonoscopy | *Gastroenterology* | Urban G; et al | 2018 | 341 | 55 |
| Real-time differentiation of adenomatous and hyperplastic diminutive colorectal polyps during analysis of unaltered videos of standard colonoscopy using a deep learning model | *GUT* | Byrne MF; et al | 2019 | 328 | 44 |
| Computer-aided tumor detection in endoscopic video using color wavelet features | *IEEE Transactions on Information Technology in Biomedicine* | Karkanis SA; et al | 2003 | 314 | 6 |
| Automatic Multi-Organ Segmentation on Abdominal CT With Dense V-Networks | *IEEE Transactions on Medical Imaging* | Gibson E; et al | 2018 | 305 | 4 |
| [Real-Time Use of Artificial Intelligence in Identification of Diminutive Polyps During Colonoscopy](https://www.acpjournals.org/doi/full/10.7326/M18-0249): A Prospective Study | *Annals of Internal Medicine* | MORI Y; et al | 2018 | 249 | 130 |
| Automated Polyp Detection in Colonoscopy Videos Using Shape and Context Information | *IEEE Transactions on Medical Imaging* | Tajbakhsh N; et al | 2016 | 242 | 3 |
| Development and validation of a deep-learning algorithm for the detection of polyps during colonoscopy | *[Nature Biomedical Engineering](https://www.nature.com/natbiomedeng)* | Wang P; et al | 2018 | 232 | 130 |
| Accurate Classification of Diminutive Colorectal Polyps Using Computer-Aided Analysis | *Gastroenterology* | CHEN PJ; et al | 2018 | 220 | 14 |
